# Heterogeneous competing causes of death and transportability of the PREVENT equations

**DOI:** 10.1016/j.ajpc.2026.101713

**Published:** 2026-06-20

**Authors:** Rebecca Arden Harris, Warren B. Bilker, Sean Hennessy

**Affiliations:** a Department of Family Medicine and Community Health, Perelman School of Medicine, University of Pennsylvania, Philadelphia, PA, USA; b Penn Cardiovascular Institute, University of Pennsylvania, Philadelphia, PA, USA; c Leonard Davis Institute of Health Economics, University of Pennsylvania, Philadelphia, PA, USA; d Department of Biostatistics and Epidemiology, University of Pennsylvania, Philadelphia, PA, USA; e Center for Real-World Effectiveness and Safety of Therapeutics, Department of Biostatistics, Epidemiology and Informatics, Perelman School of Medicine, University of Pennsylvania, Philadelphia, PA, USA

**Keywords:** Cardiovascular risk prediction, Competing risks, PREVENT equations, Calibration, Transportability, Health equity, Cause-specific mortality

## Abstract

The American Heart Association's PREVENT equations represent a major advance in cardiovascular risk prediction, in part because they account for the possibility that patients may die from non-cardiovascular causes before experiencing a cardiovascular event. Despite this advance, recent external validations have found that PREVENT systematically underestimates cardiovascular risk in Black patients, socioeconomically disadvantaged groups, veterans, and those with Medicaid or no insurance.

We propose that one underrecognized contributor is how PREVENT handles non-cardiovascular death: by treating all such deaths from cancer, overdose, suicide, injury, and other causes as a single combined category. This aggregation is a problem because the mix of competing causes differs across populations. The direct consequence is that the model's competing-risk adjustment reflects the cause-of-death composition of its derivation cohort rather than that of the population being evaluated, and standard recalibration methods cannot correct this mismatch. The direction of the resulting error is predictable: populations with disproportionately high overdose, suicide, and injury mortality relative to the cancer-dominant pattern of general-population derivation cohorts will have their cardiovascular risk systematically underestimated.

This problem is further obscured by a common methodological choice in validation studies: using Kaplan-Meier survival estimates rather than cause-specific competing-risk methods to measure observed event rates. This approach inflates apparent event rates in exactly the subgroups most affected, making miscalibration harder to interpret and potentially masking its true direction.

Future cardiovascular risk prediction models should separate non-cardiovascular deaths into cause-specific competing processes. External validations should use cause-specific competing-risk estimation. Health systems considering adoption of PREVENT should validate it locally before implementation, with particular attention to calibration within subgroups defined by competing-mortality burden.

## 1 Introduction

The American Heart Association PREVENT equations represent a major update to cardiovascular risk prediction in more than a decade, expanding beyond atherosclerotic cardiovascular disease to include heart failure, incorporating kidney function, and accounting for competing non-cardiovascular mortality [8,16]. As PREVENT becomes integrated into guideline-based treatment thresholds, accurate calibration across diverse populations is essential for equitable implementation of preventive therapies.

A key advance of PREVENT over the earlier American Heart Association/American College of Cardiology Pooled Cohort Equations (PCEs) is its treatment of non-cardiovascular death as a competing event rather than as independent censoring, addressing systematic overprediction of CVD incidence in older and multimorbid patients [8]. However, recent external validations have identified persistent subgroup-specific calibration failures across racial, ethnic, and socially disadvantaged populations despite this modern competing-risk framework and recalibration efforts [7,12,13].

One underrecognized explanation is that non-cardiovascular death may not represent a single transportable competing process. In PREVENT, all non-CVD deaths are modeled as one competing event category, but cancer, pulmonary, suicide, overdose, and injury deaths differ substantially in epidemiology and in their relationships to cardiovascular risk factors. Aggregating these causes embeds mixture weights into the competing hazard, weights that reflect the derivation population’s cause-of-death composition rather than a universal biological constant, and that baseline recalibration cannot correct. The general principle that lumping heterogeneous events into a single competing category reduces transportability of the competing hazard estimate is grounded in the competing-risk literature [2,15]; what has not been recognized is that this principle may apply directly to PREVENT and contribute to its observed calibration patterns.

## 2 The competing risk problem PREVENT solved, and the one it did not

PREVENT addresses the independent-censoring problem of the PCEs by using cause-specific hazard models that estimate each hazard separately and combine them to estimate cumulative incidence [3]: a genuine advance. What it does not resolve is heterogeneity within the competing-risk category. The derivation paper specifies that “non-CVD deaths were treated as competing events” without disaggregating by cause [8].

Aggregation is not inherently wrong: if the aggregate competing hazard accurately represents the population at hand, no bias follows. The problem arises when PREVENT is applied to populations whose non-CVD mortality composition differs from the derivation cohorts, such as veterans or socioeconomically disadvantaged groups in whom overdose, suicide, and injury deaths are disproportionately common [9,18].

Each cause has a distinct relationship to the PREVENT predictor set. Cancer shares some predictors with cardiovascular disease (smoking, diabetes, obesity) but operates through different pathways. Suicide and overdose are strongly associated with psychiatric comorbidity and social deprivation, only partially captured by PREVENT’s optional Social Deprivation Index. Pulmonary and infectious deaths are disproportionately associated with smoking and renal impairment. Age and frailty are shared predictors across most of these causes and are well captured by PREVENT; this partially attenuates aggregation error in strata where the age structure of competing causes resembles the derivation mixture.

The problem is structural: the aggregate non-CVD competing hazard can be written as a weighted sum of cause-specific hazards, *λ_ncvd_(t)* = *Σ_k_ w_k_λ_k_(t)*, where the mixture weights *w_k_* reflect the cause-of-death composition of the derivation cohort. When those weights differ across populations, the aggregate competing hazard changes even if the cause-specific hazards *λ_k_(t)* are unchanged. Recalibrating the baseline hazard shifts the intercept of the survival function but cannot restore the latent mixture weights *w_k_*: a mixture-dependent slope cannot be corrected by shifting the intercept. This limitation applies equally to slope or intercept-plus-slope recalibration, which rescales the spread of the risk score rather than the age- and time-varying shape of the competing hazard.

Independent of the recalibration question, the clinical stakes of this shift in *w_k_* are not uniform across age. In younger patients, where CVD hazard substantially exceeds non-CVD hazard, the cumulative incidence function (CIF) is relatively insensitive to competing-hazard shifts, and aggregation error may be mathematically small. In older and multimorbid patients, precisely those with the highest non-CVD mortality burden, the competing hazard carries greater weight in the CIF, and composition-driven misspecification has larger potential impact. The bias is thus largest where it matters most clinically. This mechanism predicts specific directional consequences: populations with disproportionately high external-cause and overdose mortality relative to cancer, such as veterans, Black patients, and socioeconomically disadvantaged groups, should show underprediction of CVD risk, while populations with cancer-dominant non-CVD mortality, as may occur in some Asian subgroups, should show overprediction.

## 3 Evidence from external validation studies

Two recent large validation studies illustrate both sides of the problem. Mathew and colleagues validated PREVENT in 2.5 million US veterans using cause-specific cumulative incidence estimation [13]. Yet all non-CVD deaths were modeled as a single category, consistent with the derivation. They found good overall calibration (slope 1.09) but persistent underprediction in non-Hispanic Black veterans (slope 1.29), a gap that recalibration only partially corrected. The finding is consistent with the possibility that methodologically careful validation may not fully resolve calibration gaps when the underlying cause aggregation is carried forward from the derivation.

Hong and colleagues validated PREVENT in 406,230 patients at Duke University Health System using Kaplan–Meier survival estimates rather than competing-risk estimation [7]. Kaplan–Meier estimators inflate observed cumulative incidence when competing mortality is high [10], and this overestimation is greatest in subgroups with the highest non-CVD mortality burden: Black patients, those in the most disadvantaged neighborhood quartiles, and those with Medicaid or no insurance. Recalibration improved performance only modestly; adding race, social determinants, and their interactions did not materially change results. Panel B of Figure 2 in Hong et al. illustrates the extent of this miscalibration: calibration ratios for Black patients, the most socioeconomically disadvantaged quartiles, and Medicaid-insured patients fell well below 1.0, indicating substantial underprediction of CVD risk in precisely the subgroups predicted to be most affected by competing-cause composition differences [7].

Other validations similarly used Kaplan–Meier methods [4,6], and at least one that added a Fine–Gray sensitivity analysis still treated all non-coronary deaths as a single category [11], illustrating how addressing one methodological problem while leaving the other unresolved perpetuates the limitation. Scheuermann and colleagues provide something close to a natural control condition [17]. They used Fine–Gray competing-risk methods for observed outcomes [5], and their National Health and Nutrition Examination Survey (NHANES) cohort’s non-CVD mortality composition was cancer-dominant, reasonably approximating the general-population derivation mixture. They found only modest overall miscalibration (calibration slope 1.13), with no dramatic directional subgroup failures of the kind observed by Mathew or Hong. This pattern, correct methods with a composition-matched population, is compatible with the competing-cause mechanism, though it cannot be taken as direct evidence for it.

The recalibration limitation deserves particular emphasis as the most technically robust point in this argument. Marginal recalibration adjusts the overall incidence level but cannot recover the latent mixture weights *w_k_* [15]. As noted above, this means subgroup-specific calibration errors that track competing-mortality burden persist after overall recalibration, precisely the pattern Mathew and colleagues observed.

## 4 Direction of bias by population

The directional predictions of the competing-cause mechanism are consistent with observed calibration patterns across validation studies. The observed bidirectional pattern (overprediction in some Asian subgroups, underprediction in Black patients [1,7,13,20]) is consistent with this mechanism, though unmeasured population-specific CVD risk factors remain a competing explanation. Both mechanisms are plausible and not mutually exclusive; empirical testing with disaggregated competing events is needed to estimate each contribution.

## 5 Why this matters for clinical adoption

PREVENT is now embedded in clinical guidelines, including the 2025 AHA/ACC hypertension guidelines, which use PREVENT estimates to set treatment eligibility thresholds [19]. Because the direction of miscalibration depends on the specific competing-mortality composition, some populations may be systematically overtreated while others are undertreated. Black patients and patients with Medicaid or no insurance are disproportionately represented among subgroups with the highest non-CVD mortality burdens, the population characteristic that, under the competing-cause mechanism, drives systematic miscalibration. When threshold-based guidelines determine statin eligibility or antihypertensive initiation, directional miscalibration does not distribute randomly; it clusters in populations already carrying the greatest cardiovascular burden [21]. Addressing the competing-cause aggregation problem is therefore not only a methodological refinement; it is a precondition for equitable implementation of PREVENT-based guidelines.

## 6 What would be required to address this

Addressing this requires disaggregating non-CVD deaths into cause-specific competing processes, but full disaggregation need not be the starting point. Even a two-category split for non-CVD deaths, cancer versus non-cancer, would capture the largest source of composition variation and would be feasible in most derivation cohorts, since cancer deaths are well coded and sufficiently common to support stable hazard estimates.

Critically, cause-specific competing-risk methods are needed at both stages of the evidence cycle: in the derivation sample, to estimate transportable cause-specific competing hazards, and in external validation, to place predicted and observed cumulative incidence on the same methodological footing. Addressing one without the other leaves the transportability problem only partially resolved.

Health systems contemplating adoption of PREVENT should validate the model in their own patient population before implementation. Local validation should assess calibration both overall and within subgroups defined by competing-mortality burden, including race and ethnicity, insurance status, and neighborhood deprivation, using cause-specific competing-risk estimation for observed outcomes. Without cause-of-death data, a health system cannot determine whether poor calibration reflects composition-driven competing-cause mismatch, systematic CVD risk factor differences, or both; nor can it determine the extent to which recalibration will correct the problem versus leaving subgroup-specific errors intact. This diagnostic information is a prerequisite for deciding whether PREVENT can be adopted as derived, requires local recalibration, or needs structural modification.

Cause-of-death information is available in most PREVENT derivation cohorts, and ICD coding allows coarse cause-specific decomposition in administrative datasets. One practical consideration is cause-of-death misclassification: death certificates may carry errors, with concordance varying by cause [14]. Re-derivation with disaggregated competing events would benefit from validation using linked clinical records or adjudicated endpoints where available.

## 7 Conclusion

PREVENT represents a genuine advance in cardiovascular risk prediction. The concern raised here is that aggregating all non-CVD deaths into a single competing category introduces a transportability vulnerability whose mechanism is specific: the aggregation embeds mixture weights *w_k_* that baseline recalibration cannot correct, because recalibration shifts the intercept while those weights remain fixed. Whether this accounts for a clinically meaningful share of observed calibration failures requires direct empirical testing, particularly given that age and frailty, shared predictors across competing causes, may partially attenuate the error in some strata. This uncertainty is compounded by the frequent use of Kaplan–Meier methods in external validation studies, which inflates observed cumulative incidence and may obscure the true direction and magnitude of miscalibration. Naming both mechanisms clearly is the first step toward that investigation.

## Funding

RAH is supported by K23DA054157.

## Disclosures

None.

## CRediT authorship contribution statement

**Rebecca Arden Harris:** Conceptualization, Writing – original draft. **Warren B. Bilker:** Writing – review & editing. **Sean Hennessy:** Writing – review & editing.

## Declaration of competing interest

The authors declare that they have no known competing financial interests or personal relationships that could have appeared to influence the work reported in this paper.
